# Free-breathing late gadolinium enhancement CMR with a fixed short scan time using CosMo

**DOI:** 10.1186/1532-429X-14-S1-O21

**Published:** 2012-02-01

**Authors:** Mehdi H Moghari, Hussein Rayatzadeh, Susie Hong, Raymond H Chan, Mehmet Akcakaya, Beth Goddu, Lois A Goepfert, Kraig V Kissinger, Warren J Manning, Reza Nezafat

**Affiliations:** 1 Harvard Medical School, Cambridge, MA, USA; 2 Medicine, Beth Israel Deaconess Medical Center, Boston, MA, USA

## Summary

To evaluate the performance of compressed sensing for motion correction (CosMo) [1] in compensating the respiratory motion of the heart in 3D late gadolinium enhancement (LGE) CMR.

## Background

Diaphragmatic navigators are used to reduce the artifacts caused by the respiratory motion of the heart, but this approach prolongs scan acquisition time by a factor of 2-3 [2]. Long scan times impede acquisition of high resolution LGE and degrade image quality due to contrast washout and imperfect inversion time [3].

## Methods

CosMo was implemented on a Philips 1.5T CMR system to prospectively acquire high resolution undersampled k-space LGE. The inner 10% of k-space is acquired within a 7 mm gating window. The rest of k-space is acquired without gating to finish the scan in a fixed time. Retrospectively, the k-space data acquired outside the gating window is discarded to generate the undersampled k-space. The discarded k-space is estimated using compressed sensing [4,5] to generate the images. In vivo studies were conducted on 10 healthy subjects (6 female, 30.1 ± 16.8 yr) and 9 patients (6 male, 56.8 ± 11.7 yr). The 3D LGE were acquired using the CosMo and following imaging parameters: TE/TR/α = 2.4 ms/5.2 ms/25°, spatial resolution of 1.7×1.7×1.7 mm^3^. The images were graded by consensus of two expert readers in terms of diagnostic value, based on a 2-point scale (1-yes; 2-no), and respiratory motion artifacts, based on a 4-point scale (1-severe; 4-none).

## Results

**Figure 1:**
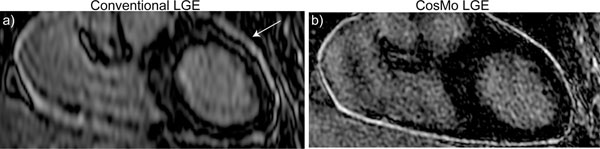
Reformatted LGE images of a suspected pericarditis patient acquired with the conventional LGE (a) and LGE with CosMo (b). LGE with CosMo reduces scan acquisition time allowing for increasing the spatial resolution.

**Figure 2:**
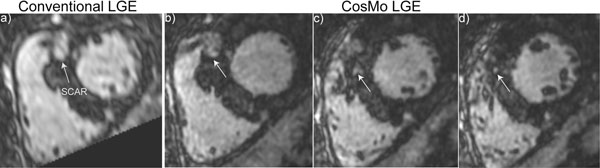
Reformatted LGE images of a patient acquired with the conventional LGE (a) and LGE with CosMo (b,c,d). LGE with CosMo reduces scan acquisition time allowing for increasing the spatial resolution.

Figure 1 demonstrates reformatted LGE images acquired from a suspected pericarditis patient using the conventional LGE and CosMo. Figure 2 displays reformatted LGE images acquired from a patient using the conventional LGE and CosMo. Although the scan time of both acquisitions was identical (≈8min.), the through-plane resolution of CosMo LGE was higher than the conventional (4.0 mm vs. 1.7 mm). The size and morphology of enhancements in the high resolution CosMo LGE is more localized compared with the conventional acquisition due to less partial volume averaging. All images acquired from 19 volunteers using the CosMo LGE were identified as diagnostic with a respiratory motion artifact score of 3.1 ± 0.7, and gating efficiency of 94 ± 4%.

## Conclusions

3D LGE with CosMo allows for reduced scan time and isotropic spatial resolution.

## Funding

NIH.
